# microRNA-17-5p Modulates *Bacille Calmette-Guerin* Growth in RAW264.7 Cells by Targeting ULK1

**DOI:** 10.1371/journal.pone.0138011

**Published:** 2015-09-18

**Authors:** Xiangguo Duan, Tao Zhang, Shuqin Ding, Jun Wei, Chunxia Su, Hongpeng Liu, Guangxian Xu

**Affiliations:** 1 School of LaboratoryMedicine, Ningxia Medical University, Yinchuan, 750004, China; 2 General Hospital of Ningxia Medical University, Yinchuan, 750004, China; 3 Ningxia Key Laboratory of Clinical and Pathogenic Microbiology, Yinchuan, 750004, China; 4 Taian City Central Hospital,Taian City, Shandong Province, 271000, China; University of Saarland Medical School, GERMANY

## Abstract

To explore the potential roles of miRNAs in controlling the survival of mycobacteria in macrophages, miR-17-5p in the regulation of Bacillus Calmette-Guérin(BCG)growth in the macrophage RAW264.7 cells was interrogated. Our results reveal that an infection of BCG shows a time-dependent up-regulation of miR-17-5p in RAW264.7 cells in early phase; importantly, excessive expression of miR-17-5p in these cells exhibits an increased propagation of intracellular BCG. Mechanistically, the Unc-51 like autophagy activating kinase 1 (ULK1), an initial molecular of autophagy are identified as novel target of miR-17-5p, the miR-17-5p is capable of targeting down-regulating the expression of ULK1 protein. In addition, an overexpression of miR-17-5p in RAW264.7 cells is correlated with repression of ULK1 and the autophagosome related proteins LC3I/II. These results imply that miR-17-5p may be able to arrest the maturation of *mycobacterial* phagosomes in part by targeting ULK1, subsequently reduces the ability of host cells to kill intracellular BCG.

## Introduction

MicroRNAs (miRNAsor miR) are evolutionarily conserved, endogenous, single-stranded, non-coding RNA molecules with approximately of **~**22 nt length of which function as post transcriptional regulators by pairing to the 3' untranslated region (UTR) of target mRNAs, subsequently inhibit the translations of mRNA [1,2,3]. An increasing number of studies have recently demonstrated that miRNAs play a regulatory role in immune responses of host cells against a pathogen invasion by targeting an immune-related signaling pathway, including the Toll-like receptor (TLR), mitogen-activated protein kinase (MAPK) and nuclear factor kappa B (NF-κB) signaling pathways [4,5,6,7]; however, an activation of targeted gene of an miRNA or its signaling cascade could in turn alter the miRNA expression in host cells [8,9,10,11,12,13]. These studies suggest that the miRNAs and inflammatory signaling molecules can create a fine-tuned feedback loop to regulate immune responses in hosts.

The miR-17-5p belongs to the cluster miR-17-92 or miR-17 family which encodes six miRNAs (miR-17-5p, miR-18a, miR-19a, miR-20a, miR-19b-1, and miR-92a-1) is able to bind the same seed sequence of 3'-UTRs of their targeted genes. The miR-17-92 cluster has shown functions of oncogenes by regulating cell proliferation, apoptosis and development [14,15]. Owing to an increasingly appreciated importance of miRNA families, the functions of miR-17-92 cluster have been intensively characterized, by which these miRNAs have shown an essential role in tumorigenesis and normal development of organs including the heart, lungs, and immune system [16,17,18,19], and regulation of immune response to an pathogen infection [20,21].

At the same time, another studies have revealed that the abundances of miR-17~92 cluster of host cells can be altered upon an intracellular pathogen infection, by which a pathogen may gain its intracellular resistance to be eliminated by the host cell through an apoptotic or autophagic cell death [22,23,24,25]. However, the underlying mechanism between miRNAs and phagosomes of the pathogen-host interaction remains elusive. As all known, in the case of a mycobacterial infection, *Mycobacterium tuberculosis* (*M*. *tuberculosis*, Mtb) and *M*. *bovis*bacilleCalmette-Guerin (BCG) can be ingested by alveolar macrophage and reside within phagosomes, in which they resist acidification and fusion with lysosomes by altering the maturation of the phagosomes [26].

Interestingly, a recent study on human glioblastoma cells demonstrated that the miR-17-5p was able to inhibit cell autophagy by targeting ATG7 and inhibiting the fusion of phagosomes and lysosomes [27], implying the miR-17-5p may play a regulatory role in lysosomal maturation.

Therefore, we herein explore the potential role of miR-17-5p in alveolar macrophages in response to BCG infection, we found that the infection of BCG induces a up-regulation of miR-17-5p in macrophage RAW264.7 cells in early phase, and inhibition of miR-17 transcript increases the abundance of ULK1 and LC3I/II protein, and improves the capacity to kill the intracellular bacilli in macrophages.

## Materials and Methods

### Cells culture and transfection

This study was approved by the ethics committee of the Ningxia Medical University (Permit Number 2014–135).

Murine macrophage RAW264.7 cells (ATCC; TIB-71) and human HEK 293T cells were purchased from the Type Culture Collection of the Chinese Academy of Sciences, Shanghai, China. The cells were cultured and maintained at 37°C in a humidified atmosphere of 5% CO_2_ 95% oxygen, RPMI 1640 and DMEM medium (Gibco) supplemented with 10% Fetal Bovine Serum (FBS) and 1% pen/strep. For a transfection of oligonucleotides, the oligonucleotides of miRNA mimics or inhibitor were transfected into RAW264.7 cells using Lipofectamine 2000 reagents per manufacturer’s instructions (Invitrogen, USA). For cells cultured in a 6-well plate, 20 μM of oligonucleotides was transfected.

### miR-17-5p expression in RAW264.7 cells with BCG infection

The macrophage RAW264.7 cells were infected with BCG at an MOI of 10 for the indicated time points and the miR-17-5p transcript was determined by a qRT-PCR assay.

*Mycobacterium bovis* BCG Beijing strain was purchased from the Center for Disease Control and Prevention (CCDC) of china (Beijing, China). The bacilli were grown at 37°C with shaking in Middle-brook 7H9 broth containing 10% albumin dextrose catalase supplement for 2 weeks, the bacterial cultures were then harvested by centrifugation at 500×g for 10 min and the cell pellets were resuspended in the BCG culture medium. The number of viable colony-forming units (CFU) of the culture was determined by plating serially diluted cultures on Middlebrook 7H11 plates supplemented with OADC enrichment (BD Biosciences Shanghai, Shanghai, China), and the bacterial colonies were counted after 4 weeks of culture [28]. Aliquots of the stock were stored at -80°C. Cells were infected with BCG at a multiplicity of infection (MOI) of 10 and incubated at 37°C in a 5% CO_2_, humidified air atmosphere for indicated time before they were harvested for analysis.

### Generation of plasmid expressing miR-17-5p

In order to construct a vector expressing miR-17-5p, oligonucleotides of sense strand (5’-TCAAAGTGCTTACAGTGCAGGTAGTTCAAGAGACTACCTGCACTGTAAGCACTTTGTTTTTTC-3’) and antisense strand (5’-TCGAGAAAAAACAAAGTGCTTACAGTGCAGGTAGTCTCTTGAACTACCTGCACTGTAAGCACTTTGA-3’) were synthesized, which were based on the sequence of mouse miR-17-5p (5’-caaagtgcttacagtgcaggtag-3’, MIMAT0000649) from miRBase database. Appropriate sites of restriction endonucleases were also introduced at 5’-ends of these oligonucleotides. The mixture of the sense and anti-sense oloigonucleotides was then used for the production of the precursor of small hairpin RNA (shRNA) of miR-17-5p by temperature annealing approach. The miR-17 precursor was modified with appropriate restriction enzymes, and cloned into a miRNA expressing plasmid, pSicoR (Department of Biological Chemistry, School of Medicine, Fudan University, Shanghai, China) to generate the vector expressing miR-17-5p, which was designated as pSicoR/miR-17-5p in this study. A negative control vector was named pSicoR/NC.

### Production of lentiviral vector and infection of RAW264.7 macrophage

For production of the lentiviral vector, 1×10^6^ HEK 293T cells were seeded per well in six-well plates with 2 mL of DMEM/10% FBS without antibiotics. The next day, the medium was replaced with 1 mL DMEM without FBS and antibiotics. Subsequently, the pSicoR/miR-17-5p vector (1.5μg) or pSicoR/NC (1.5μg) was co-transfected with packaging plasmids pCMV-VSV-G (0.5μg) and pCMV-dR8.91 (1μg) (Department of Biological Chemistry, School of Medicine, Fudan University, Shanghai, China) with TransLipid Transfection Reagent as suggested by the manufacturer. The medium was replaced with 2 mL of DMEM/10% FBS at 6 h post transfection. The supernatant was harvested at 48 h after transfection, followed by being filtered through a 0.45-μm size filter, and then concentrated to 1/100 volume by ultracentrifugation with 50000× g at 4°C for 2.5 h using a SW28 rotor (Beck-man Coulter, Fullerton, CA, USA) and Sorvall Ultra 80® (Kendro Laboratory Products, Newtown, CT, USA). The virus particle pellet was resuspended in PBS and frozen at -80°C till use. The viral particles were titrated in 293T cells by counting EGFP-positive cells.RAW264.7 cells were infected with vector-containing supernatant directly after 1 day in culture, which was designated as LV-miR-17-5p or LV-NC. The cells were infected with a lentiviral vector at MOI of 20 in this study.

### Dual-luciferase reporter vector containing ULK1 3’UTR sequence for validation of miR-17-5p target

In order to validate the *ULK1* gene were the targets of miR-17-5P, the reporter plasmids containing luciferase with the 3’UTR sequence of murine *ULK1*mRNA were generated. The primers used for amplification of wild-type (WT) and mutated (Mut) 3’UTR of ULK1mRNA were designed based on GenBank database (J05287.1). The sequences were as follow: The forward primers for ULK1/Mut ULK1 3′UTR: 5'-CGACGCGTCGTATGGTGCCGAGAAGTGG-3', the reverse primers for ULK1/Mut ULK1: 5'-CGAGCTCGGAGGACGAAAGCCAGGAA-3'/5'-CGAGCTCGTAACGTACTCCGGTGACT-3'. The restriction sites (underlined) of SacI and Mlu I were respectively introduced in the forward and reverse primers. The cDNA generated from RAW264.7 RNA was used as templates for amplification of the 3’UTR fragment by a PCR assay. The wild-type and mutated 3’UTR fragment were then cloned into the downstream of luciferase reporter gene of pMIR-Report vector (Promega, Madison, WI, USA), by which the ULK1 mRNA luciferase reporter vectors, pMIR-Report/ULK1 (harboring WT 3’UTR) and pMIR-Report/Mut-ULK1 (containing a Mut 3’UTR) were generated. The specificity of miR-17-5p targeting ULK1 mRNA was ascertained by co-transfection of pSicoR/miR-17-5P and pMIR-ReportULK1 or pMIR-Report/Mut-ULK1 into 293T cells, and determined by the relative activity of firefly luciferase unit (RLU) at 48 h post-transfection using a dual-luciferase reporter assay kit as recommended by the manufacturer (Promega, Madison, WI, USA). A Renilla luciferase expressing plasmid pRL-TK (Promega, Madison, WI, USA) was always included in the transfection to normalize the efficiency of each transfection [29,7].

### Immunoblotting analysis

To obtain total cellular lysates, the cells were lysed in an ice-cold cell lysis buffer from a protein extraction kit. The protein concentration was determined with the Bradford protein assay kit (BESTBIO) using a c-globulin standard curve. Proteins were resolved by standard SDS-PAGE and transferred onto PVDF membranes (Millipore, USA). Nonspecific binding sites were blocked using 5% dry skimmed milk, 0.2% Tween-20 in PBS (pH 7.4) for overnight at 4°C and then incubated with primary antibodies againstULK1、 LC3I/II and GAPDH (Cell Signaling Technology, Inc, Danvers, MA, USA). The membranes were then incubated at room temperature (RT) for 1 h with appropriate HRP-conjugated secondary antibodies prior to be developed using the enhanced Western Bright ECL reagent (Advansta, United States).

### Immunocyto-fluorescent staining for confocal microscopy

The RAW264.7 cells cultured on a cover slide were transfected with miR-17-5p mimic or miR-17-5p inhibitor for 24 h, and then treated with rapamycin or BCG and different design for 2 h before they were used for immunocyto-fluorescent analysis. The cells were first fixed in 4% paraformaldehyde in PBS at RT for 20 min prior to be permeabilized by PBS containing 0.2% Triton X-100 for 10 min. Followed by blocking with PBS containing 3% bovine serum albumin (BSA) before the slide was serially incubated with rabbit anti-ULK1 andanti- LC3I/II (1:200 dilution) at 4°C for over night and then Rhodamine-conjugated goat anti-rabbit IgG antibody (1:200 dilution) at RT for 1 h. The slides were then rinsed with PBS and mounted with Prolong Gold anti-fade reagent with 4’-6-diamidino-2-phenylindole (DAPI) (P36931, Invitrogen, Grand Island, NY, USA). The images of cells were visualized and acquired using an Olympus DSU spinning disk confocal microscope under a 100 × objective oil lens. The relative expression of ULK1 and LC3I/II in the cells was ascertained by quantifying the fluorescent signal of Rhodamine using ImageJ Software version 1.46 (http://rsb.info.nih.gov/ij/). Fold change was calculated as the ratio between the relative intensity of each sample over a control sample.

### qPCR examination for BCG burdened in RAW264.7 cells

The RAW264.7 cells were infected with LV-NC, LV-miR-17-5p, or transfected with miR-17-5p inhibitor or control oligonucleotides for 24 hours. Then the intracellular BCG was quantified by a qPCR assay. Briefly, the cells were transfected with the pSicoR/miR-17-5p or miR-17-5p inhibitor and incubated for additional 24 h prior to be infected with BCG for indicated time. The cells were then harvested for total DNA extraction using a commercially available genomic DNA kit (TIANGEN BIOTECH,Beijing,China). The abundance of intracellular bacilli was determined by accessing BCG DNA using a qPCR assay; the primer set used for qPCR was based on BCG specific sequence (IS6110, Sequence ID: gi|6006564|emb|X57835.2|); the sequences of primers were 5’-GGACGGAAACTTGAACACG-3’ (forward) and 5’-TCTGACGACCTGATGATTGG-3’ (reverse); standard PCR cycle parameters were as follows: 95°C for 30 s, followed by 40 cycles of 95°C for 15 s and 60°C for 30 s and 72°C for 30 s. The BCG abundance was calculated against the β-actin, an internal control of host cells, using a comparative Ct (ΔΔCt) method.

### Bioinformatic analysis

The putative targets of miR-17-5p were predicted using the miRanda, PicTar and TargetScan target algorithms.

### Statistical Analysis

Statistical analyses were performed using two-tailed Student’s *t-test*. Comparisons between groups were performed using ANOVA. Significant differences were assigned to *p* values <0.05 and <0.01, and denoted by * and **, respectively. Data was presented as the mean ± standard deviation (SD).

## Results

### BCG inhibits miR-17-5p expression in RAW264.7 cells

**Fig 1:**
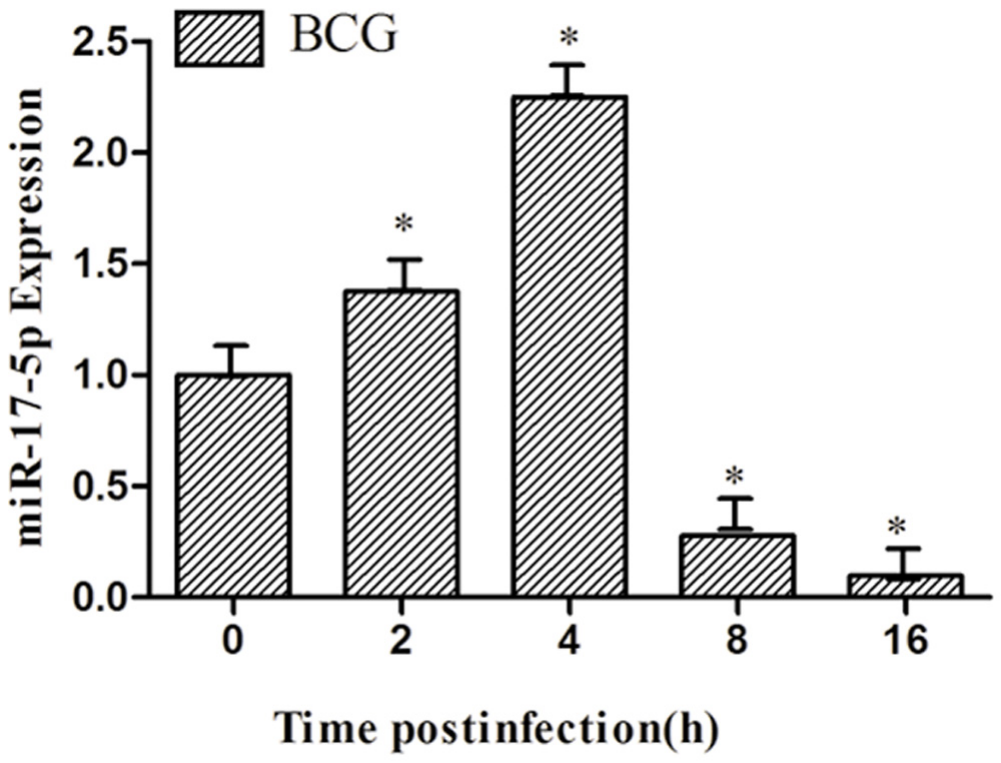
A time-dependent up-regulation of miR-17-5p expression in macrophages upon a BCG infection. A time-dependent manner of miR-17-5p was observed in RAW264.7 cells following the BCG infection. Data are expressed as the mean ± SD of three independent experiments (N = 9). Compared to the uninfected cells, *: p<0.05.

The result showed that an infection of BCG induced an up-regulation of miR-17-5p expression in a time-dependent manner (Fig 1). This data implies that the miR-17-5p may play a regulatory role in macrophages in response to mycobacterial infection.

### Construction of lentiviral vector and infection of RAW264.7 macrophage

**Fig 2:**
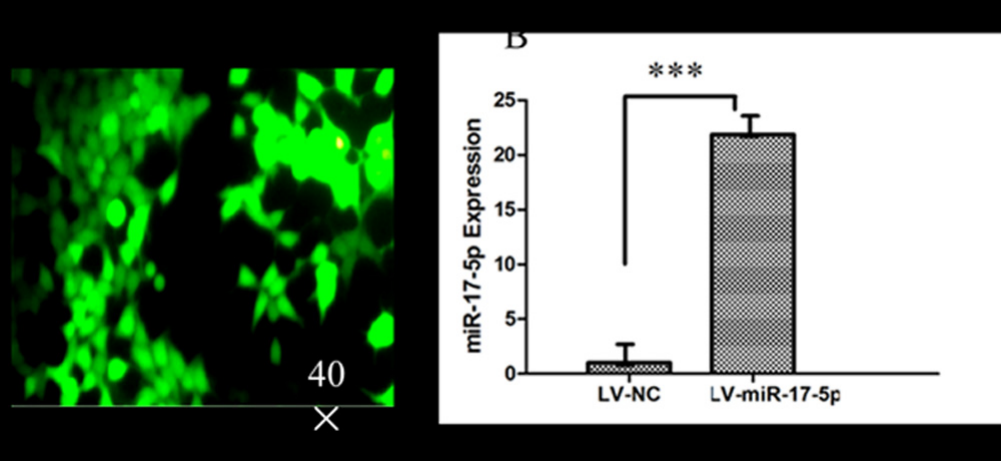
The generation of lentiviral vector expressing miR-17-5p. A. The transfection of LV-miR-17-5p in RAW264.7 cells by Fluorescent microscopy. B. miR-17-5p expression from LV-miR-17-5p/ LV-NCinfected RAW264.7 cells by qRT-PCR. Data represents the mean ± SD from three independent experiments. ***: p<0.001.

Lentiviral vectors expressing miR-17-5p (LV-miR-17-5p) and control (LV-NC) were successfully constructed, and the viral particles were titrated in 293T cells by accessing the EGFP-positive cell colonies (Fig 2A). The titers of viruses were 1x10^8^ TU/mL. In addition, the miR-17-5p transcript in RAW264.7 cells transduced with LV-miR-17-5p was significantly abundant relative to the cells infected with LV-NC (p<0.05) (Fig 2B), indicating a functional viral vector was generated.

### miR-17-5p inhibit the expression of ULK1 by targeted ULK1–3´UTR

**Fig 3:**
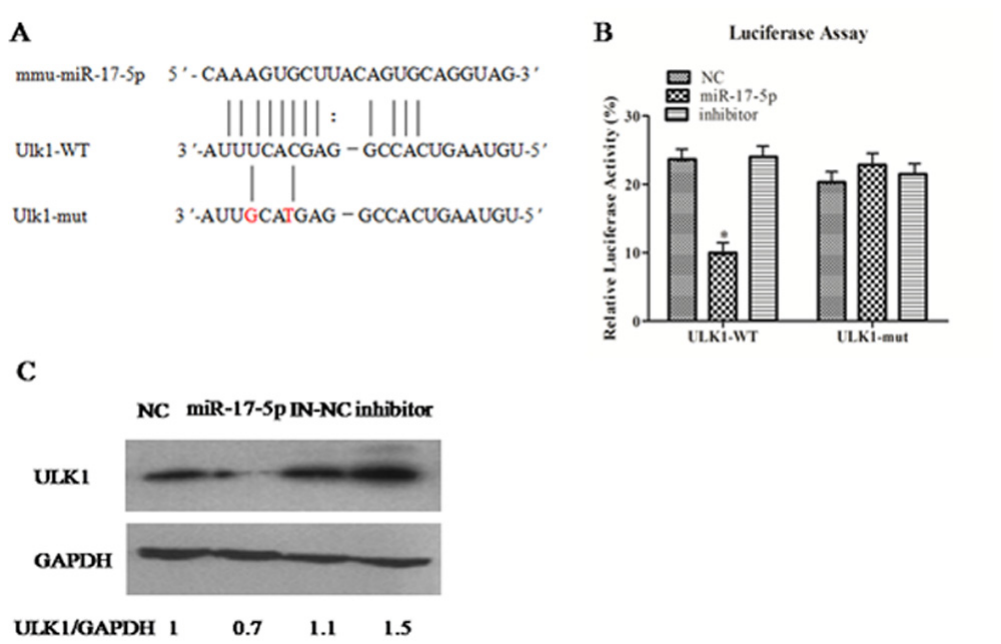
miR-17-5p inhibit the expression of ULK1 by targetedULK1–3´UTR. A. Identification of the miR-17-5p target genethrough the informatics software; B. Identification of miR-17-5p target ULK1 by dual luciferase assay; C. Identification of miR-17-5p target ULK1by Western-blot. Data are shown as the mean ± SEM of three independent experiments. *: p<0.05; Values of ULK1/GAPDH ratios are indicated below the representative blot.

To identify the target gene of the miR-17-5p with ULK1 through the informatics software, we found the binding site as(Fig 3A); dual luciferase data shows that the miR-17-5p can obviously inhibit the luciferase activity of ULK1-3’UTR, but not the luciferase activity of ULK1-3’UTR mutant, (Fig 3B); our data shows that the miR-17-5p can obviously inhibit the expression of ULK1 protein by western-blot(Fig 3C).

### The influence of BCG to miR-17-5p modulate the expression of ULK1

**Fig 4:**
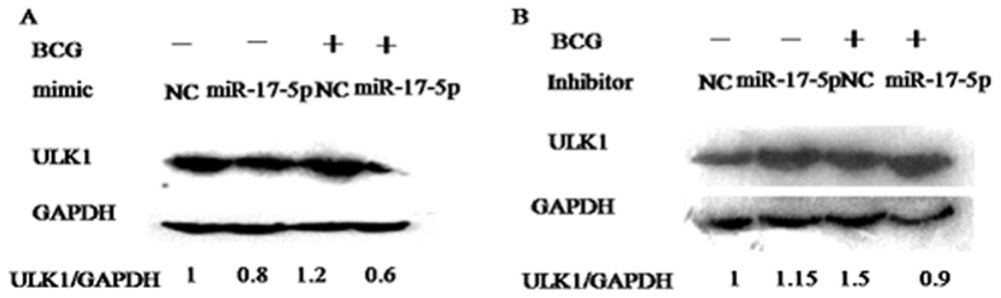
The decrease of ULK1 expression by miR-17-5p from BCG infection. A. ULK1 expression from miR-17-5p mimic plus BCG by Western-blot. B. ULK1 expression from miR-17-5pinhibitor plus BCG by Western-blot. Data are shown as the mean ± SEM of three independent experiments. *: p<0.05.

The results showed that the expression of ULK1from themiR-17-5p mimics plus BCG infection group obviously was decreased compared with NC plus BCG (Fig 4A), On the contrary, the expression of ULK1 from the miR-17-5p inhibitor group obviously was increased (Fig 4B).

### The influence of BCG to miR-17-5p can obviously inhibit the occurrence of autophagy

**Fig 5:**
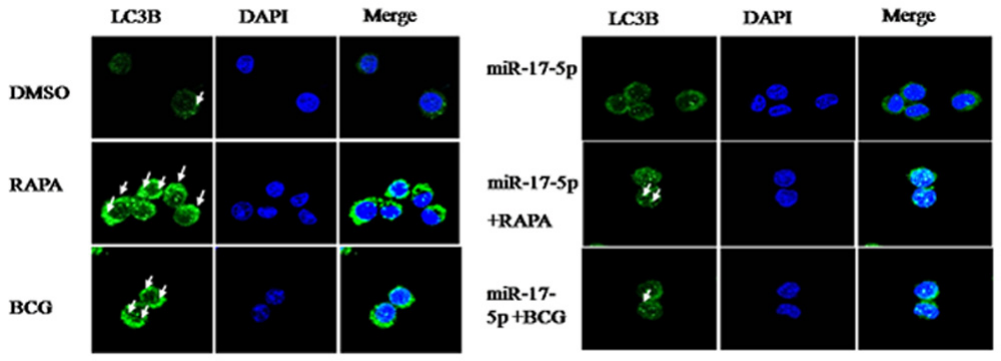
The detection of the Autophagical protein LC3 by Confocal Microscopy. LC3II antibody followed by Alexa Fluor 488-conjugated goat anti-rabbit IgG (Green). Nuclear stain DAPI (Blue).

The data shows that the Rapamycin can obviously induce the occurrence of autophagy but not BCG compare with DMSO (Fig 5), and the miR-17-5p mimic plusBCG can inhibit the occurrence of autophagy compare with miR-17-5p mimic (Fig 5). All together data shows that the influence of BCG tomiR-17-5p can obviously inhibit the occurrence of autophagy.

### miR-17-5p modulate the expression of LC3B

**Fig 6:**
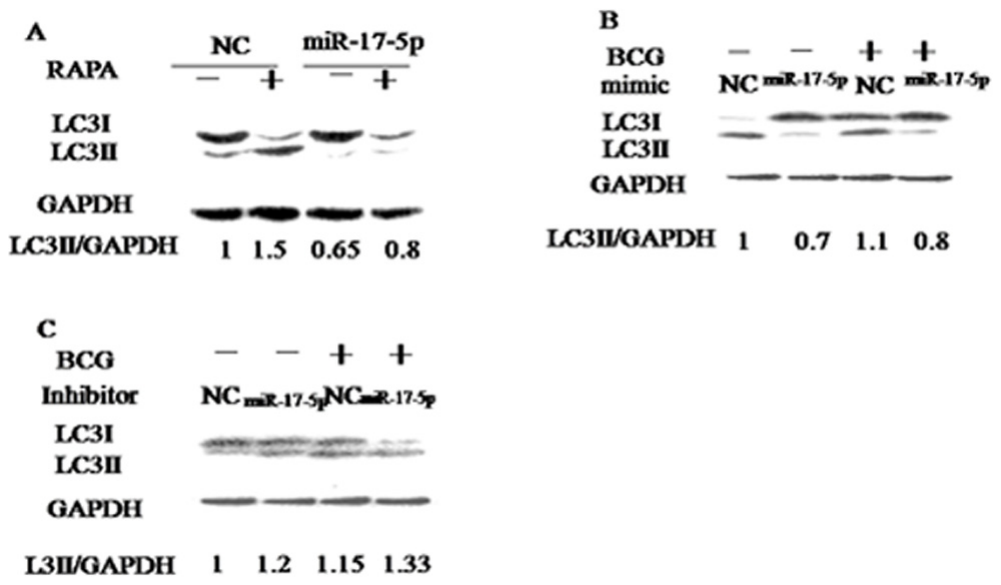
The expression of LC3II/I from miR-17-5p mimic/inhibitor plus BCG or RAPAby Western blot. A. miR-17-5p plus RAPA. B. miR-17-5p mimic plus RAPA or BCG. C. miR-17-5p inhibitor plus RAPA or BCG. Data are shown as the mean ± SEM of three independent experiments. *: p<0.05. Values of LC3II/GAPDH ratios are indicated below the representative blot.

The data shows that the ratio of LC3II/I fromRapamycin group obviously was increased compared with normal control, but the ratio of LC3II/I from miR17-5p group obviously was decreased compared with NC group (Fig 6A). Similarly, the ratio of LC3II/I from the BCG plus miR-17-5p mimicgroup was alsodecreased compared with BCG plus NC(Fig 6B), but the ratio of LC3II/I from the BCG plus miR-17-5p inhibitor group was obviously increased compared with the BCG plus NC(Fig 6C). Therefore, we conclude that the miR-17-5p can modulate the BCG induced cyto-autophagy.

### miR-17-5p increases the growth of BCG in RAW264.7 cells by qPCR

**Fig 7:**
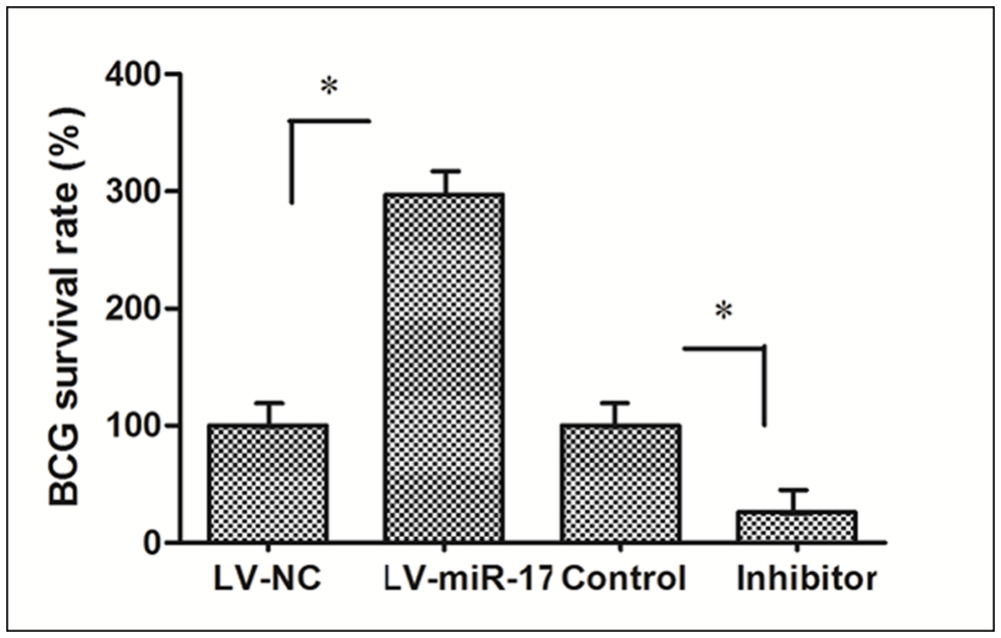
miR-17-5p promotes BCG growth in RAW264.7 cells. *: p<0.05. Data represents the mean ± SD from three independent triplicated experiments (N = 9).

An abundant BCG was detected fromLV-miR-17-5p group compared with LV-NC, less abundant BCG was determined from miR-17-5p inhibitor compared to the controls(Fig 7). Therefore,miR-17-5p increases the growth of BCG in RAW264.7 cells.

## Discussion

*Mycobacterium tuberculosis* (Mtb)is one of the most harmful intracellular pathogens, which is the cause of Tuberculosis (TB), a devastating infectious disease worldwide [30]. It is therefore important to understand the underlying mechanisms of pathogenesis and immunological responses required for protection hosts against TB infection [31]. Alveolar macrophages are main targets of Mtb infection, and the pattern of cell death of Mtb-infected alveolar macrophages has been recognized to play a central role in the pathogenesis of tuberculosis (TB) [32].

Mounting evidence has corroborated that phagocytosis of invaded Mtb by macrophages plays a pivotal role in the innate immune response to Mtb infection, particularly in a latent Mtb infection of macrophages [33,34,35]. In this context, the invaded pathogens are ingested into phagosomal vacuoles of which they interact with endosomal and lysosomal vesicles through a process of phagolysosome biogenesis, during which phagosomes gain properties degradation and microbicide to kill and degrade the phagocytozed pathogens [36]. However, an Mtb has an ability to prevent phagosome-lysosome fusion and arrest the maturation of phagosomes, subsequently acquires a capacity to survive and proliferate within phagosomes in macrophages by inhibiting phagolysosome biogenesis [37,38]. To date, the precise mechanism by which Mtb arrests pathogen-phagocytozedphagolysosome maturation remains elusive, but recent studies found that Mtb-loaded phagosomes within dendritic cells were correlated with lysosomes in the early stages of infection [39]; Previous studies in intracellular *T*. *gondii* infection have also suggested that miR-17~92 cluster played a regulatory role in the innate apoptotic response of macrophages [22,23]; in addition, both of miR-17-5p and miR-30d, have been found to be able to inhibit the maturation of lysosomes containing mycobacteria through a mechanism of regulation of specific genes related to lysosomal maturation and/or a mechanism of autophagy [27,40]. In line with these findings, we found that an infection of BCG can induce a time-dependent up-regulation of miR-17-5p in macrophage RAW264.7 cells; on the contrary, an increasing level of miR-17-5p transcript in the cells by a lentiviral transduction exhibits an increased intracellular BCG in RAW264.7 cells as compared with the uninfected cells. Mechanistically, in part, the miR-17-5p isable to target ULK1 mRNA and suppress ULK1 and LC3I/II protein expression, subsequently may arrests the maturation of BCG phagosomes. As a consequence, the ability to kill BCG in the host cells is decreased, which thereby leads an increased growth of BCG in the cells.

As the main targets of Mtb infection, alveolar macrophages are able to provide critical intracellular niches for Mtb establishing infection in host [32]. In our report, we show that miR-17-5p is able to suppressand the initial autophagosome protein ULK1 and LC3I/II expression and promote BCG growth in macrophages partly by a mechanism of inhibition phagosomal maturation. This result supports the notion of that ULK1 and LC3I/II are the critical molecular for the fusions of autophagosomes and lysosomes in a chaperone-mediated autophagy of mammalian cells [41]. However, the detailed underlying mechanism of the ULK1 and LC3I/II-mediated autophagy needs to be further explored in future.

Collectively, we interrogated the biological function of miR-17-5p in macrophages in response to mycobacterial infection. We found that the miR-17-5p were able to directly bind to the 3’-UTR of ULK1 mRNA and inhibit ULK1 and LC3I/II protein expression post-transcriptionally; a BCG infection could increase the miR-17-5p transcript in RAW264.7 cells in a time-dependent manner; intriguingly, an excessive expression of miR-17-5p in RAW264.7 cells showed a tendency to promote BCG growth in the host cells. These results thus implicated a previous undefined mechanism by which the miR-17-5p is able to arrest the maturation of BCG phagosomes, accordingly reduces the ability to kill BCG in the host cells. This finding also highlights an underlying mechanism by which miR-17-5p modulates phagosomal maturation in a mycobacterial latent infection.

## References

[1] Amano A, Nakagawa I, Yoshimori T (2006) Autophagy in innate immunity againstintracellular bacteria. Journal of biochemistry 140, 161–6. 10.1093/jb/mvj162 16954534

[2] Chen K, Song F, Calin GA, Wei Q, Hao X, Zhang W (2008) Polymorphisms in microRNA targets: a gold mine for molecular epidemiology. Carcinogenesis 29, 1306–1311. 10.1093/carcin/bgn116 Epub 2008 May 13. 18477647

[3] Majoros WH, Ohler U (2007) Spatial preferences of microRNA targets in 3' untranslated regions. BMC genomics 8, 152 10.1186/1471-2164-8-152 17555584 PMC1904200

[4] Foley NH, O'Neill LA (2012) miR-107: a toll-like receptor-regulated miRNA dysregulated in obesity and type II diabetes. Journal of leukocyte biology 92, 521–7. 10.1189/jlb.0312160 22645244

[5] Gaziel-Sovran A, Segura MF, Di Micco R, Collins MK, Hanniford D, Vega-Saenz de Miera E, et al (2011) miR-30b/30d regulation of GalNAc transferases enhances invasion and immunosuppression during metastasis. Cancer cell 20, 104–18. 10.1016/j.ccr.2011.05.027 21741600 PMC3681522

[6] Wei J, Huang X, Zhang Z, Jia W, Zhao Z, Zhang Y, et al (2013) MyD88 as a target of microRNA-203 in regulation of lipopolysaccharide or Bacille Calmette-Guerin induced inflammatory response of macrophage RAW264.7 cells. Molecular immunology 55, 303–9. 23522925 10.1016/j.molimm.2013.03.004

[7] Xu G, Zhang Z, Wei J, Zhang Y, Zhang Y, Guo L, et al (2013) microR-142-3p down-regulates IRAK-1 in response to Mycobacterium bovis BCG infection in macrophages. Tuberculosis (Edinburgh, Scotland) 93, 606–11. 10.1016/j.tube.2013.08.006 24053976

[8] Bazzoni F, Rossato M, Fabbri M, Gaudiosi D, Mirolo M, Mori L, et al (2009) Induction and regulatory function of miR-9 in human monocytes and neutrophils exposed to proinflammatory signals. Proceedings of the National Academy of Sciences of the United States of America 106, 5282–7. 19289835 10.1073/pnas.0810909106 PMC2664036

[9] Liu G, Friggeri A, Yang Y, Park YJ, Tsuruta Y, Abraham E (2009) miR-147, a microRNA that is induced upon Toll-like receptor stimulation, regulates murine macrophage inflammatory responses. Proceedings of the National Academy of Sciences of the United States of America 106, 15819–24. 10.1073/pnas.0901216106 19721002 PMC2747202

[10] Ma C, Li Y, Li M, Deng G, Wu X, Zeng J, et al (2014) microRNA-124 negatively regulates TLR signaling in alveolar macrophages in response to mycobacterial infection. Molecular immunology 62, 150–158. 24995397 10.1016/j.molimm.2014.06.014

[11] O'Connell RM, Kahn D, Gibson WS, Round JL, Scholz RL, Chaudhuri AA, et al (2010) MicroRNA-155 promotes autoimmune inflammation by enhancing inflammatory T cell development. Immunity 33, 607–19. 10.1016/j.immuni.2010.09.009 20888269 PMC2966521

[12] O'Neill LA, Sheedy FJ, McCoy CE (2011) MicroRNAs: the fine-tuners of Toll-like receptor signalling. Nature reviews. Immunology 11, 163–75. 10.1038/nri2957 21331081

[13] Taganov KD, Boldin MP, Chang KJ, Baltimore D (2006) NF-kappaB-dependent induction of microRNA miR-146, an inhibitor targeted to signaling proteins of innate immune responses. Proceedings of the National Academy of Sciences of the United States of America 103, 12481–6. 10.1073/pnas.0605298103 16885212 PMC1567904

[14] He L, Thomson JM, Hemann MT, Hernando-Monge E, Mu D, Goodson S, et al (2005) A microRNA polycistron as a potential human oncogene. Nature 435, 828–33. 10.1038/nature03552 15944707 PMC4599349

[15] Mendell JT (2008) miRiad roles for the miR-17-92 cluster in development and disease. Cell 133, 217–22. 18423194 10.1016/j.cell.2008.04.001 PMC2732113

[16] Baumjohann D, Kageyama R, Clingan JM, Morar MM, Patel S, de Kouchkovsky D, et al (2013) The microRNA cluster miR-17 approximately 92 promotes TFH cell differentiation and represses subset-inappropriate gene expression. Nature immunology 14, 840–8. 10.1038/ni.2642 Epub 2013 Jun 30. 23812098 PMC3720769

[17] Cox MB, Cairns MJ, Gandhi KS, Carroll AP, Moscovis S, Stewart GJ, et al (2010) MicroRNAs miR-17 and miR-20a inhibit T cell activation genes and are under-expressed in MS whole blood. PloS one 5, e12132 20711463 10.1371/journal.pone.0012132 PMC2920328

[18] Kang SG, Liu WH, Lu P, Jin HY, Lim HW, Shepherd J, et al (2013) MicroRNAs of the miR-17 approximately 92 family are critical regulators of T(FH) differentiation. Nature immunology 14, 849–57. 10.1038/ni.2648 23812097 PMC3740954

[19] Shan SW, Lee DY, Deng Z, Shatseva T, Jeyapalan Z, Du WW, et al (2009) MicroRNA MiR-17 retards tissue growth and represses fibronectin expression. Nature cell biology 11, 1031–8. 10.1038/ncb1917 19633662

[20] Philippe L, Alsaleh G, Pichot A, Ostermann E, Zuber G, Frisch B, et al (2013) MiR-20a regulates ASK1 expression and TLR4-dependent cytokine release in rheumatoid fibroblast-like synoviocytes. Annals of the rheumatic diseases 72, 1071–9. 10.1136/annrheumdis-2012-201654 23087182

[21] Poitz DM, Augstein A, Gradehand C, Ende G, Schmeisser A, Strasser RH (2013) Regulation of the Hif-system by micro-RNA 17 and 20a—role during monocyte-to-macrophage differentiation. Molecular immunology 56, 442–51. 23911400 10.1016/j.molimm.2013.06.014

[22] Cai Y, Chen H, Jin L, You Y, Shen J (2013) STAT3-dependent transactivation of miRNA genes following Toxoplasma gondii infection in macrophage. Parasites & vectors 6, 356 10.1186/1756-3305-6-356 24341525 PMC3878672

[23] Cai Y, Chen H, Mo X, Tang Y, Xu X, Zhang A, et al (2014) Toxoplasma gondii inhibits apoptosis via a novel STAT3-miR-17-92-Bim pathway in macrophages. Cellular signalling 26, 1204–12. 24583285 10.1016/j.cellsig.2014.02.013

[24] Zeiner GM, Norman KL, Thomson JM, Hammond SM, Boothroyd JC (2010) Toxoplasma gondii infection specifically increases the levels of key host microRNAs. PloS one 5, e8742 10.1371/journal.pone.0008742 20090903 PMC2806928

[25] Zhu D, Pan C, Li L, Bian Z, Lv Z, Shi L, Zhang J, et al (2013) MicroRNA-17/20a/106a modulate macrophage inflammatory responses through targeting signal-regulatory protein alpha. The Journal of allergy and clinical immunology 132, 426–36.e8. 10.1016/j.jaci.2013.02.005 23562609 PMC5882493

[26] Xu S, Cooper A, Sturgill-Koszycki S, van Heyningen T, Chatterjee D, Orme I, et al (1994) Intracellular trafficking in Mycobacterium tuberculosis and Mycobacterium avium-infected macrophages. Journal of immunology 153, 2568–78. 8077667

[27] Comincini S, Allavena G, Palumbo S, Morini M, Durando F, Angeletti F, et al (2013) microRNA-17 regulates the expression of ATG7 and modulates the autophagy process, improving the sensitivity to temozolomide and low-dose ionizing radiation treatments in human glioblastoma cells. Cancer biology & therapy 14, 574–86. 10.4161/cbt.24597 23792642 PMC3742487

[28] Lewin A, Freytag B, Meister B, Sharbati-Tehrani S, Schafer H, Appel B (2003) Use of a quantitative TaqMan-PCR for the fast quantification of mycobacteria in broth culture, eukaryotic cell culture and tissue. Journal of veterinary medicine. B, Infectious diseases and veterinary public health 50, 505–9. 10.1046/j.1439-0450.2003.00715.x 14720189

[29] Ma C, Li Y, Zeng J, Wu X, Liu X, Wang Y (2014) Mycobacterium bovis BCG triggered MyD88 induces miR-124 feedback negatively regulates immune response in alveolar epithelial cells. PloS one 9, e92419 10.1371/journal.pone.0092419 24705038 PMC3976256

[30] Arnvig K, Young D (2012) Non-coding RNA and its potential role in Mycobacterium tuberculosis pathogenesis. RNA biology 9, 427–36. 22546938 10.4161/rna.20105 PMC3384566

[31] Modlin RL, Bloom BR (2013) TB or not TB: that is no longer the question. Science translational medicine 5, 213sr6 24285487 10.1126/scitranslmed.3007402

[32] Lee J, Repasy T, Papavinasasundaram K, Sassetti C, Kornfeld H (2011) Mycobacterium tuberculosis induces an atypical cell death mode to escape from infected macrophages. PloS one 6, e18367 10.1371/journal.pone.0018367 21483832 PMC3069075

[33] Bruns H, Stegelmann F, Fabri M, Dohner K, van Zandbergen G, Wagner M, et al (2012) Abelson tyrosine kinase controls phagosomal acidification required for killing of Mycobacterium tuberculosis in human macrophages. Journal of immunology 189, 4069–78. 10.4049/jimmunol.1201538 22988030 PMC3684563

[34] Seto S, Tsujimura K, Horii T, Koide Y (2013) Autophagy adaptor protein p62/SQSTM1 and autophagy-related gene Atg5 mediate autophagosome formation in response to Mycobacterium tuberculosis infection in dendritic cells. PloS one 8, e86017 10.1371/journal.pone.0086017 24376899 PMC3871604

[35] Steinhauser C, Dallenga T, Tchikov V, Schaible UE, Schutze S, Reiling N (2014) Immunomagnetic isolation of pathogen-containing phagosomes and apoptotic blebs from primary phagocytes. Current protocols in immunology / edited by John E. Coligan…[et al] 105, 14.36.1–14.36.26. 10.1002/0471142735.im1436s105 24700322

[36] Martin CJ, Booty MG, Rosebrock TR, Nunes-Alves C, Desjardins DM, Keren I, et al (2012) Efferocytosis is an innate antibacterial mechanism. Cell host & microbe 12, 289–300. 10.1016/j.chom.2012.06.010 22980326 PMC3517204

[37] Clemens DL, Horwitz MA (1995) Characterization of the Mycobacterium tuberculosis phagosome and evidence that phagosomal maturation is inhibited. The Journal of experimental medicine 181, 257–70. 7807006 10.1084/jem.181.1.257 PMC2191842

[38] Deretic V, Singh S, Master S, Harris J, Roberts E, Kyei G, et al (2006) Mycobacterium tuberculosis inhibition of phagolysosome biogenesis and autophagy as a host defence mechanism. Cellular microbiology 8, 719–27. 10.1111/j.1462-5822.2006.00705.x 16611222

[39] Van der Wel N, Hava D, Houben D, Fluitsma D, van Zon M, Pierson J, et al (2007) M. tuberculosis and M. leprae translocate from the phagolysosome to the cytosol in myeloid cells. Cell 129, 1287–98. 10.1016/j.cell.2007.05.059 17604718

[40] Yang X, Zhong X, Tanyi JL, Shen J, Xu C, Gao P, et al (2013) mir-30d Regulates multiple genes in the autophagy pathway and impairs autophagy process in human cancer cells. Biochemical and biophysical research communications 431, 617–22. 10.1016/j.bbrc.2012.12.083 23274497 PMC3578012

[41] Dohi E, Tanaka S, Seki T, Miyagi T, Hide I, Takahashi T, et al (2012) Hypoxic stress activates chaperone-mediated autophagy and modulates neuronal cell survival. Neurochemistry international 60, 431–42. 10.1016/j.neuint.2012.01.020 22306777

